# Influence of Tiapride and Topiramate on Tic Severity and Behavioral/Emotional Problems in Children with Tourette Syndrome: A Retrospective Study

**DOI:** 10.3390/brainsci13030383

**Published:** 2023-02-22

**Authors:** Xuanzi Zhou, Zhaoying Liao, Yi Li, Nanqing Wang, Nong Xiao

**Affiliations:** 1 Department of Rehabilitation, Children’s Hospital of Chongqing Medical University, National Clinical Research Center for Child Health and Disorders, Ministry of Education Key Laboratory of Child Development and Disorders, Chongqing 400010, China; 2 China International Science and Technology Cooperation Base of Child Development and Critical Disorders, Chongqing 400010, China; 3 Chongqing Key Laboratory of Translational Medical Research in Cognitive Development and Learning and Memory Disorders, Chongqing 400010, China

**Keywords:** Tourette syndrome, tiapride, topiramate, tics, behavioral problems, emotional problems

## Abstract

Objectives: To investigate the effect of tiapride and topiramate on patients with Tourette syndrome (TS). Methods: This retrospective analysis included 126 children diagnosed with TS at Children’s Hospital of Chongqing Medical University from 2019 to 2021, with treatment including tiapride (n = 60) and topiramate (n = 66). Their tic severity values were assessed with the Yale Global Tic Severity Scale (YGTSS). Furthermore, behavioral and emotional problems were assessed with the Conner’s Parent Rating Scale (CPRS) and the Children Behavior Checklist (CBCL). Results: Compared with premedication, the scores of tic severity were significantly decreased in both tiapride and topiramate groups after treatment, especially topiramate. Moreover, it was noted that five subscores of CPRS were significantly reduced in TS patients thanks to medication. However, there was no significant difference in CBCL after treatment, in both tiapride and topiramate groups. Conclusions: Tiapride and topiramate were proven to be effective on tics and some behavioral/emotional problems in TS patients, and topiramate may provide better treatment.

## 1. Introduction

Tourette syndrome (TS), also known as Gilles de la Tourette syndrome (GTS), was named after French neurologist Gilles de la Tourette, who first reported on the syndrome in detail in 1885. TS is now considered a neurobehavioral disorder characterized by multiple motor and vocal tics, usually starting during childhood and often accompanied by a variety of behavioral comorbidities, such as attention-deficit/hyperactivity disorder (ADHD), obsessive-compulsive disorder (OCD), poor impulse control, and other behavioral problems [1]. It has considerable repercussions for family relationships, social life, and the ability to function at work. According to the *Diagnostic and Statistical Manual of Mental Disorders, Fifth Edition* (*DSM-5*) [2], there is no diagnostic test for TS, and the diagnosis is based on a medical history and physical examination performed by a healthcare clinician [3]. The symptoms of TS begin in childhood, and the estimated prevalence is 3–9/1000. The literature states that the maximum in the severity of TS manifestations occurs at the age of 10–12 years. TS is more common in boys than in girls, with a prevalence of 3–4:1 [4]. Yang et al. reported that the prevalence of TS in China was 0.3%, which was lower than the global prevalence, and the ratio of male to female prevalence ranged from 2.17 to 10.6 [5].

In addition to ADHD and OCD, the predominant co-occurring clinical comorbid conditions in TS include obsessive-compulsive behavior (OCB) and autism spectrum disorder (ASD), whereas the most common coexistent psychopathologies include depressive illnesses, depressive symptomatology, learning difficulties, personality disorders, oppositional defiant disorders, and conduct disorders [6]. In addition, antisocial behavior, inappropriate sexual activity, exhibitionism, aggressive behavior, discipline problems, sleep disturbances, and self-injurious behaviors are found in a substantial percentage of TS patients [7]. Although largely unknown, evidence is emerging for etiological links between these comorbid disorders and TS at the level of the neural substrate, suggesting that comorbidity is a central feature of the syndrome [8]. The symptoms of TS and the associated comorbid conditions may interact and result in negative experiences and poor quality of life.

When the tics are mild to moderate, many TS patients do not require pharmacological treatment. When the tics are severe and cause problems with speech or activities, then behavioral therapy or medication should be considered. A variety of drugs (typical and atypical antipsychotics, alpha adrenergic agonists, dopamine depletors, benzodiazepines, and botulinum toxins) are commonly used in the treatment of tics and other symptoms connected with TS. Tiapride is the first-line treatment of TS in Europe, for its few side effects. Topiramate is an antiepileptic drug that is also used as a mood stabilizer. Some studies have shown topiramate to be effective for TS treatment. Tics are often reduced by medication, but there are few studies on the effects of medication on patients’ behavior. Thus, the aim of this study was to investigate whether pharmacological treatment has an effect on the behavior of TS patients.

## 2. Materials and Methods

### 2.1. Data Sources

Outpatients who visited the neurorehabilitation department at the Children’s Hospital of Chongqing Medical University from 2019 to 2021 were eligible for inclusion in the trial after being diagnosed with TS by a pediatrician on the basis of *DSM-5* criteria, which is as follows: (1) Both multiple motor and one or more vocal tics have been present at some time during the illness, although not necessarily concurrently. (2) The tics may wax and wane in frequency but have persisted for more than 1 year since first tic onset. (3) Onset is before the age of 18 years. (4) The disturbance is not attributable to the physiological effects of a substance (e.g., cocaine) or another medical condition (e.g., Huntington’s disease, postviral encephalitis). TS children and their parents gave consent to medication treatment. All patients completed the Yale Global Tic Severity Scale (YGTSS), Conner’s Parent Rating Scale (CPRS), and the Children Behavior Checklist (CBCL) before and after taking medication. Furthermore, the inclusion criteria for TS children were as follows: no intellectual impairments, no drug-induced involuntary movements, and no diagnosed neurological disorders. In this study, children who had previously been taking medications but later discontinued treatment for any reason or had accepted other medications or treatments were excluded. This retrospective study was approved by the ethics committee of the Children’s Hospital of Chongqing Medical University.

### 2.2. Data Collection

We collected basic information on TS children through the outpatient system, including gender, age, and follow-up time. The YGTSS, CPRS, and CBCL results of the corresponding patients were collected at T1 (premedication) and T2 (follow-up) in the assessment room.

### 2.3. Instruments

#### 2.3.1. Yale Global Tic Severity Scale (YGTSS)

The YGTSS is a semistructured instrument of motor and vocal tic severity [9]. It is administered by a trained clinician to assess symptomology over the previous week. Ratings are assessed for motor and vocal tics on five domains each: number, frequency, intensity, complexity, and interference. A separate, one-item impairment rating is also included. The scores from each dimension (number, frequency, intensity, complexity, and interference) are summed to produce the total motor tic score (range 0–25), the total vocal tic score (range 0–25), and the combined total tic score (range 0–50). The scale also includes a separate impairment scale (range 0–50). Finally, the global severity score is a summation of the total motor tic score, the total vocal tic score, and the impairment score.

#### 2.3.2. Conner’s Parent Rating Scale (CPRS)

The CPRS was used to measure ADHD symptom severity in the last 4 weeks, as assessed by children’s parents. The foremost function of the CPRS will be as a screening tool or as an adjunctive instrument to a comprehensive assessment [10]. The other major use of the CPRS is to monitor treatment and to assess treatment outcome. It comprises 48 items, consisting of 6 subscores: conduct problems, learning problems, psychosomatic disorders, impulsivity-hyperactivity, anxiety, and ADHD index. Ratings on the questionnaire ranged from 0 (never or rarely) to 3 (very often).

#### 2.3.3. Children Behavior Checklist (CBCL)

The CBCL [11] was used to obtain standardized reports from parents on behavioral and emotional problems in their children. It contains 113 problem items and measures internalizing and externalizing problems experienced by the child. It was also used to measure total problems experienced by the child, as well as the child’s total competence [12]. Parents rate each item as 0 for not true, 1 for somewhat or sometimes true, and 2 for very true or often true, on the basis of the behavior of their children. The ratings on various constellations of the CBCL items are added to produce raw scores for each of 9 behavioral syndromes.

### 2.4. Statistical Analysis

The software IBM SPSS Statistics version 23.0 (IBM Corp., Armonk, NY, USA) was used for data analysis, and the significance level was set to *p* < 0.05. In this study, the outcomes were tics and behaviors, which measured the score at follow-up time over the baseline (premedication score) for each patient. The descriptive statistics, including the totals, proportions, and frequencies, were obtained from the categorical and ratio variables.

We used a chi-square test to demonstrate whether a significant relationship existed between the topiramate group (TPM) and the tiapride hydrochloride group (tiapride) in gender. A *t* test was conducted to examine the equality of the variables among age and follow-up time within the TPM/tiapride groups.

The TPM values of the instruments were compared with tiapride group values, respectively, using a *t* test to analyze whether there were significant changes in tics and the other behaviors of the TPM/tiapride groups. Post-treatment instruments were compared with baseline (pretreatment), with paired *t*-tests to analyze whether there were significant changes pre-/post-treatment.

## 3. Results

### 3.1. Patient Group Characteristics

In total, 129 eligible children were enrolled in the study. There were two patients in the tiapride group and one in the TPM group lost upon follow-up. Their demographic information and clinical characteristics are summarized in Table 1. The TPM and tiapride groups included 66 subjects with an average age of 8.2 (SD = 2.3) and 60 subjects with an average age of 8.2 (SD = 2.1), respectively. The clinical characteristics, including gender, age, and follow-up time, were not significantly different between the TPM and tiapride groups (*p* > 0.05, TPM vs. tiapride, Table 1).

### 3.2. Changes in Tic Severity and Impairment between the TPM and Tiapride Groups

As seen in Table 2, there was a significant difference in tic severity and impairment after both TPM and tiapride treatments (*p* < 0.05) compared with premedication. Before the treatments, there was no significant difference in tic severity and impairment values between TPM and tiapride. The TPM group had significantly lower total severity scores than the tiapride group did (*p* < 0.01). Moreover, the TPM group had significantly lower YGTSS scores for all components (motor, vocal, and impairment) than the tiapride group did. In summary, TPM treatment significantly improved the treatment outcomes for patients with TS compared with patients treated with tiapride.

### 3.3. Changes in Behavior between the TPM and Tiapride Groups

Table 3 shows the results of the one-way ANOVA for scores of the CPRS. In T1, there was no significant difference in CPRS values between TPM and tiapride. However, compared with the tiapride group, the conduct problems, learning problems, impulsivity-hyperactivity, anxiety, and ADHD index values for behavior significantly decreased in T2 in the TPM group. Moreover, there was a significant difference in the above five subscores after both TPM and tiapride treatments compared with premedication, while the comparisons of two groups in psychosomatic disorders were nonsignificant. In summary, TPM treatment significantly improved the five behaviors for patients with TS compared with patients treated with tiapride treatment.

Table 4 list the results of the CBCL of the TPM and tiapride groups. There were no significant differences in internalizing problems, social withdrawal, and externalizing problems between the TPM group and the tiapride group—neither premedication nor post-treatment (*p* > 0.05). Furthermore, the comparisons of T1 and T2 in the TPM group and the tiapride group were nonsignificant.

## 4. Discussion

This study revealed the effects of different medications on children with TS by observing the changes of tics and the other behaviors of patients with TS who were treated with TPM (n = 66) or tiapride (n = 60). Our results showed that TS was more common in boys than girls and preferentially affected boys, which was consistent with the international consensus. TS concentrated mainly in those aged 7 to 8 years, and our study showed the age of TS children is around 8.

The scores of tic severity measured by the YGTSS were significantly decreased in both the TPM and tiapride groups after treatment. Furthermore, the scores of the TPM group were compared with those of the tiapride group to identify tics in this study. The results of this comparison showed that the TPM group had significantly lowered the YGTSS scores compared with the tiapride group after treatment. Therefore, it is suggested that two kinds of pharmacological treatments can both significantly relieve tics, especially the TPM. These findings partly agree with those of previous studies. In a study of 49 pediatric patients with tic disorders(TDs) treated with tiapride showed that the symptoms improved in 83.3% of the patients [13]. However, a meta-analysis, which included 14 studies evaluating the efficacy of topiramate for the treatment of TDs, reported that compared with haloperidol and tiapride, topiramate appears to be a promising medication with good efficacy and tolerability for children with TDs [14]. Our sample also demonstrated significantly lowered impairment scores in TPM than tiapride did after treatment.

Tics are the result of the dysfunction of the basal ganglia pathway. The tics of TS patients include motor tics and vocal tics, which also need to be distinguished. Functional imaging studies of patients with Tourette syndrome using resting-state fMRIs have shown the activation of the anterior cingulate cortex and insula immediately preceding tic onset, but they have not attempted to distinguish between motor tics and vocal tics [15]. Some patients with mild symptoms require no treatment. However, in some patients, motor and vocal tics can be persistent and severe and affect social, physical, and psychological interactions. In this group, patients need treatment such as behavioral therapies and neuroleptic medication. Our findings, showing an improvement in motor tic severity and vocal tic severity following medication interventions, are consistent with recent reports regarding medication treatment for individuals with tic disorders. Similar to the previous reports, this study has demonstrated the advantage of medication intervention on tic reduction.

Our study of CPRS revealed six subscores of behavioral and emotional problems in TS children. The results of five subscores, measured by the CPRS, were significantly decreased in both the TPM and tiapride groups after treatment. Previous studies have reported that behavioral therapy can improve not only the tics of TS patients but also the patients’ behavioral problems. A single-blind, randomized controlled trial showed that online exposure response prevention (ERP) for tics also produced an improvement in anxiety after 6 months of treatment [16]. Meanwhile, a study containing 59 TS children and adolescents showed that baseline anxiety scores evaluated by the parents predicted a significant decrease after combined habit reversal training (HRT) and ERP treatment [17]. Furthermore, group-based CBIT for TDs patients showed a significant reduction in tics and the severity of comorbid symptoms such as anxiety, behavioral problems, and aggressive behaviors [18,19]. However, few studies have directly investigated the relationship between medication and comorbid symptoms in children with TS. Our study showed that the medication may improve some comorbid symptoms in TS patients.

CBCL is also a questionnaire evaluating behavioral and emotional problems in children and adolescents. However, there were no significant differences in the scores of CBCL after medication. One behavioral therapy is in line with the results of our study. In it, 47 TD children and adolescents were re-examined 1 year after manualized treatment, and the outcome demonstrated that none of the scores, namely the PUTS score, BATS score, CBCL, SCARED score, or MFQ score, differed when comparing the therapeutic patients and nontherapeutic patients at 12 months [20]. These two questionnaires may be the reason for the large standard deviation. Previous reports have examined the measurement invariance and discriminant validity of the CPRS and assessed its convergent validity against the CBCL; the estimated correlations between various scores of these two were moderately strong to very weak [21]. Another possible explanation could be the follow-up period.

Children with TS often have comorbid disorders, such as ADHD, OCD, anxiety, depression, autism spectrum disorder, sleep disorders, migraine, rage attacks, and self-injurious behavior. The study found that 86% of patients with TS had a lifetime prevalence of any psychiatric symptoms and that 58% of them had more than two psychiatric illnesses [22]. The primary nuclei of the basal ganglia are significant in motor control, emotion, and cognition, and it is also the pathway for tic production [23]; from this, we can also understand the connection between emotions and tics. A systematic review [24] found that in TS children, the comorbid symptoms can have a particularly severe impact on school life, emotions, social contact, and physical well-being that persist into adulthood. Moreover, the impact of comorbid depression and anxiety on quality of life (QoL) seems to become more apparent with age. A large interview study showed that non-tic-related symptoms can be more problematic than the tics themselves [25]. All these lead to the significance of improving comorbid disorders.

Tiapride, a benzamide agent, which acts as a selective dopamine antagonist at the dopamine D2 and D3 receptors, is recommended as first-line medication in China [26]. Tiapride has no influence on the neurosecretory, hypothalamic-hypophyseal regulation of sex hormones, the thyroid stimulating hormone, the growth hormone, or the thyroid hormone [27]. The main adverse reactions of tiapride are weight gain, drowsiness, and hyperprolactinemia. Topiramate is a sulfamate-modified fructose diacetonide, but its mechanisms of action are still unknown. Yu et al. [14] showed that topiramate is a promising medication with good efficacy and tolerability for children with TDs, compared with haloperidol and tiapride. The most common side effects for topiramate are language problems, cognitive disorders, kidney stones, and weight loss.

Finally, the limitations of this study should be considered. First, the study was retrospective, and we did not collect enough data for a nontherapeutic group. Thus, high-quality RCTs are required. Second, our study is a single-center design, which reduces the generalizability of the findings. Moreover, the sample size for this study was small, and the follow-up period was relatively short. Further research should be conducted with a larger sample size and a longer follow-up period.

## 5. Conclusions

In this study, both tiapride and topiramate were demonstrated to be effective in reducing tics in TS patients; topiramate was particularly effective in this regard. Furthermore, some behavioral and emotional problems were significantly relieved after the treatment of tiapride and topiramate. The findings of the current study are particularly important and meaningful for the treatment of TS.

## Figures and Tables

**Table 1:** TPM and tiapride group demographic and clinical characteristics.

		TPM (n = 66)	Tiapride (n = 60)	t	*p*
Gender (%)	Female	13 (19.7%)	10 (16.7%)		0.660
	Male	53 (80.3%)	50 (83.3%)		-
Age, years (Mean ± SD)		8.2 ± 2.3	8.2 ± 2.1	1.09	0.925
Follow-up time, months (Mean ± SD)		5.7 ± 3.4	5.4 ± 3.8	1.73	0.610

TPM: topiramate; Tiapride: tiapride hydrochloride; SD: standard deviation; *t* = *t* statistic, *p* = *p*-value.

**Table 2:** Comparison of the YGTSS scores between TPM group and tiapride group.

	TPM (n = 66)		Tiapride (n = 60)		t (T1)	* p * (T1)	t (T2)	*p* (T2)
	T1	T2	T1	T2				
	Mean (SD)	Mean (SD)	Mean (SD)	Mean (SD)				
Total motor tic score	11.4 (4.1) ^a^	6.1 (4.7)	12.1 (4.3) ^b^	9.4 (3.6)	−0.92	0.362	−4.08	<0.01
Total vocal tic score	6.8 (5.2) ^a^	2.3 (4.1)	7.1 (5.2) ^b^	4.8 (4.6)	−0.31	0.756	−3.22	<0.05
Impairment score	30 (7.3) ^a^	15.8 (9.4)	31.6 (7.6) ^b^	24.1 (8.9)	−2.65	0.309	−4.97	<0.01
Total severity score	49.2 (10.5) ^a^	23.9 (15.5)	50.8 (12.2) ^b^	38.2 (13.5)	−2.27	0.135	−5.40	<0.01

TPM: topiramate; Tiapride: tiapride hydrochloride; SD: standard deviation; T1: premedication; T2: follow-up; ^a^ TPM T1 vs. TPM T2, *p* < 0.05; ^b^ Tiapride T1 vs. Tiapride T2, *p* < 0.05; *p* (T1): TPM T1: tiapride T1; *p* (T2): TPM T2: tiapride T2; *t* = *t* statistic, *p* = *p*-value.

**Table 3:** Comparison of the CPRS scores between TPM group and Tiapride group.

	TPM (n = 66)		Tiapride (n = 60)		t (T1)	* p * (T1)	t (T2)	*p* (T2)
	T1	T2	T1	T2				
	Mean (SD)	Mean (SD)	Mean (SD)	Mean (SD)				
Conduct problems	2.2 (0.3) ^a^	1.4 (0.4)	2.2 (0.2) ^b^	2 (0.3)	−0.52	0.610	−8.01	<0.01
Psychosomatic disorders	0.9 (1.2)	0.6 (0.3)	0.7 (0.6)	0.7 (0.5)	0.82	0.414	−1.78	0.078
Anxiety	2.4 (0.3) ^a^	1.2 (0.2)	2.3 (0.3) ^b^	2.2 (0.3)	0.71	0.482	−16.12	<0.01
Learning problems	2.6 (0.4) ^a^	1.9 (0.4)	2.6 (0.3) ^b^	2.3 (0.4)	−0.24	0.808	−3.94	<0.01
Impulsivity-hyperactivity	2.5 (0.2) ^a^	1.3 (0.3)	2.5 (0.3) ^b^	2 (0.4)	−0.11	0.911	−8.46	<0.01
ADHD index	2.5 (0.3) ^a^	1.5 (0.4)	2.5 (0.3) ^b^	2.1 (0.4)	−0.94	0.350	−6.72	<0.01

TPM: Topiramate; Tiapride: Tiapride hydrochloride; SD: standard deviation; T1: premedication; T2: follow-up; ^a^ TPM T1 vs. TPM T2, *p* < 0.05; ^b^ Tiapride T1 vs. Tiapride T2, *p* < 0.05; *p* (T1): TPM T1: Tiapride T1; *p* (T2): TPM T2: Tiapride T2; t = t statistic, *p* = *p*-value.

**Table 4:** Comparison of the CBCL scores between TPM group and Tiapride group.

	TPM (n = 66)				Tiapride (n = 60)				t (T1)	* p * (T1)	t (T2)	*p* (T2)
	T1		T2		T1		T2					
	Mean	SD	Mean	SD	Mean	SD	Mean	SD				
Internalizing problems	44.4	5.4	43.7	4.6	46.5	4.8	46.0	4.0	−1.16	0.255	−1.42	0.148
Schizoid	7.4	1.4	7.3	1.2	8.0	1.6	8.0	1.7	−1.23	0.229	−1.69	0.102
Depressed	10.2	3.1	10.2	3.0	11.2	2.7	11.1	2.3	−0.97	0.339	−0.96	0.344
Unhealthy contact	6.4	1.8	6.4	1.8	6.0	0.0	6.0	0.0	0.80	0.429	0.80	0.429
Obsessive	10.5	1.0	10.2	0.9	11.0	0.8	10.7	0.9	−1.65	0.108	−1.57	0.126
Somatic complaints	10.0	1.3	9.7	1.4	10.3	1.4	10.1	1.2	−0.63	0.535	−0.91	0.369
Social withdrawal	6.8	1.6	6.9	1.5	6.5	1.1	6.5	1.1	0.62	0.568	0.86	0.422
Externalizing problems	41.9	3.5	41.1	3.5	43.2	3.6	43.5	3.0	−1.09	0.285	−1.75	0.091
Hyperactivity	13.1	0.8	13.2	1.0	13.2	0.7	13.6	0.8	−0.64	0.524	−1.14	0.261
Aggressive	20.8	1.2	20.1	1.0	20.8	1.3	20.7	1.3	−0.22	0.829	−1.32	0.197
Delinquent	8.1	2.6	8.0	2.5	9.2	2.4	9.2	0.5	−1.22	0.230	−1.50	0.169

TPM: topiramate; Tiapride: tiapride hydrochloride; SD: standard deviation; T1: premedication; T2: follow-up; *p* (T1): TPM T1: tiapride T1; *p* (T2): TPM T2: tiapride T2; *t* = *t* statistic, *p* = *p*-value.

## Data Availability

The data are not publicly available, due to privacy or ethics.

## References

[1] Jankovic J., Kurlan R. (2011). Tourette syndrome: Evolving concepts. Mov. Disord..

[2] American Psychiatric Association (2013). Diagnostic and Statistical Manual of Mental Disorders.

[3] Muth C. (2017). Tics and Tourette Syndrome. JAMA.

[4] Novotny M., Valis M., Klimova B. (2018). Tourette Syndrome: A Mini-Review. Front. Neurol..

[5] Yang C., Zhang L., Zhu P., Zhu C., Guo Q. (2016). The prevalence of tic disorders for children in China: A systematic review and meta-analysis. Medicine.

[6] Robertson M., Cavanna A., Eapen V. (2015). Gilles de la Tourette syndrome and disruptive behavior disorders: Prevalence, associations, and explanation of the relationships. J. Neuropsychiatry Clin. Neurosci..

[7] Cavanna A., Servo S., Monaco F., Robertson M. (2009). The behavioral spectrum of Gilles de la Tourette syndrome. J. Neuropsychiatry Clin. Neurosci..

[8] O’Hare D., Helmes E., Eapen V., Grove R., McBain K., Reece J. (2016). The Impact of Tic Severity, Comorbidity and Peer Attachment on Quality of Life Outcomes and Functioning in Tourette’s Syndrome: Parental Perspectives. Child Psychiatry Hum. Dev..

[9] Leckman J., Riddle M., Hardin M., Ort S., Swartz K., Stevenson J., Cohen D. (1989). The Yale Global Tic Severity Scale: Initial testing of a clinician-rated scale of tic severity. J. Am. Acad. Child Adolesc. Psychiatry.

[10] Conners C., Sitarenios G., Parker J., Epstein J. (1998). The revised Conners’ Parent Rating Scale (CPRS-R): Factor structure, reliability, and criterion validity. J. Abnorm. Child Psychol..

[11] Achenbach T.M. (1991). Manual for the Child Behavior Checklist/4-18 and 1991 Profile.

[12] Hoare J., Phillips N., Brittain K., Myer L., Zar H., Stein D. (2019). Mental Health and Functional Competence in the Cape Town Adolescent Antiretroviral Cohort. J. Acquir. Immune Defic. Syndr..

[13] Fekete S., Egberts K., Preissler T., Wewetzer C., Mehler-Wex C., Romanos M., Gerlach M. (2021). Estimation of a preliminary therapeutic reference range for children and adolescents with tic disorders treated with tiapride. Eur. J. Clin. Pharmacol..

[14] Yu L., Yan J., Wen F., Wang F., Liu J., Cui Y., Li Y. (2020). Revisiting the Efficacy and Tolerability of Topiramate for Tic Disorders: A Meta-Analysis. J. Child Adolesc. Psychopharmacol..

[15] Foltynie T. (2016). Vocal tics in Tourette’s syndrome. Lancet Neurol..

[16] Hollis C., Hall C., Jones R., Marston L., Novere M., Hunter R., Brown B., Sanderson C., Andrén P., Bennett S. (2021). Therapist-supported online remote behavioural intervention for tics in children and adolescents in England (ORBIT): A multicentre, parallel group, single-blind, randomised controlled trial. Lancet Psychiatry.

[17] Nissen J., Parner E., Thomsen P. (2019). Predictors of therapeutic treatment outcome in adolescent chronic tic disorders. BJPsych Open.

[18] Heijerman-Holtgrefe A., Verdellen C., van de Griendt J., Beljaars L., Kan K., Cath D., Hoekstra P., Huyser C., Utens E. (2021). Tackle your Tics: Pilot findings of a brief, intensive group-based exposure therapy program for children with tic disorders. Eur. Child Adolesc. Psychiatry.

[19] Zimmerman-Brenner S., Pilowsky-Peleg T., Rachamim L., Ben-Zvi A., Gur N., Murphy T., Fattal-Valevski A., Rotstein M. (2022). Group behavioral interventions for tics and comorbid symptoms in children with chronic tic disorders. Eur. Child Adolesc. Psychiatry.

[20] Nissen J., Carlsen A., Thomsen P. (2021). One-year outcome of manualised behavior therapy of chronic tic disorders in children and adolescents. Child Adolesc. Psychiatry Ment. Health.

[21] Fumeaux P., Mercier C., Roche S., Iwaz J., Stéphan P., Revol O. (2021). Validation of the French Version of Conners’ Parent Rating Scale-Revised, Short Form in ADHD-Diagnosed Children and Comparison with Control Children. J. Atten. Disord..

[22] Hirschtritt M., Lee P., Pauls D., Dion Y., Grados M., Illmann C., King R., Sandor P., McMahon W., Lyon G. (2015). Lifetime prevalence, age of risk, and genetic relationships of comorbid psychiatric disorders in Tourette syndrome. JAMA Psychiatry.

[23] Leisman G., Sheldon D. (2022). Tics and Emotions. Brain Sci..

[24] Evans J., Seri S., Cavanna A. (2016). The effects of Gilles de la Tourette syndrome and other chronic tic disorders on quality of life across the lifespan: A systematic review. Eur. Child Adolesc. Psychiatry.

[25] Stiede J., Alexander J., Wellen B., Bauer C., Himle M., Mouton-Odum S., Woods D. (2018). Differentiating tic-related from non-tic-related impairment in children with persistent tic disorders. Compr. Psychiatry.

[26] Liu Z., Cui Y., Sun D., Lu Q., Jiang Y., Jiang L., Wang J., Luo R., Fang F., Zhou S. (2020). Current Status, Diagnosis, and Treatment Recommendation for Tic Disorders in China. Front. Psychiatry.

[27] Roessner V., Schoenefeld K., Buse J., Bender S., Ehrlich S., Münchau A. (2013). Pharmacological treatment of tic disorders and Tourette Syndrome. Neuropharmacology.

